# Report of Basal Cell Carcinoma Occurring With a Desmoplastic Trichilemmoma Successfully Treated With Mohs Micrographic Surgery

**DOI:** 10.7759/cureus.61910

**Published:** 2024-06-07

**Authors:** Haleigh D Stafford, Yelena Dokic, Yve Huttenbach, Carina Wasko, Jennifer S Ranario

**Affiliations:** 1 School of Medicine, Baylor College of Medicine, Houston, USA; 2 Department of Dermatology, Baylor College of Medicine, Houston, USA; 3 Department of Pathology, Immunology, and Dermatopathology, Baylor College of Medicine, Houston, USA

**Keywords:** dermatopathology, dermatology and dermatologic surgery, mohs micrographic surgery, basal cell carcinoma, desmoplastic trichilemmoma

## Abstract

Desmoplastic trichilemmoma, an uncommon variant of trichilemmoma, is a benign adnexal neoplasm originating from the outer root sheath of a hair follicle, which has rarely been associated with atypical basaloid proliferations, including basal cell carcinoma. In this patient case, a 67-year-old female presented to our dermatology clinic for a skin check. On physical examination, a pearly, pink papule was noted on the vertex scalp, and a biopsy was obtained to rule out malignancy. Histologic examination of the lesion favored a desmoplastic trichilemmoma; however, a basaloid neoplasm could not be ruled out. Subsequently, the patient underwent Mohs micrographic surgery, and upon examination of the Stage I Mohs slides, superficial basal cell carcinoma was identified within the lesion. This case serves to further strengthen the known association between basal cell carcinoma and desmoplastic trichilemmoma. In addition, it demonstrates that the presence of basal cell carcinoma may not be observed on the initial biopsy of these lesions, underscoring the utility of complete surgical excision.

## Introduction

Herein, we describe a case of basal cell carcinoma occurring with a desmoplastic trichilemmoma successfully treated with Mohs micrographic surgery. Desmoplastic trichilemmoma (DT), a histologic variant of trichilemmoma, is a benign adnexal neoplasm originating from the outer root sheath of the hair follicle [1]. The incidence of DT is poorly defined; however, Crowson and Magro reported 28 cases of DT out of 120,000 skin biopsies obtained in an 18-month period [2]. Clinically, DT may present as an erythematous or skin-colored, slow-growing, asymptomatic solitary papule. The surface may be smooth, keratotic, ulcerated, or pearly and telangiectatic [3]. Consequently, histologic examination is necessary for a correct diagnosis, as DT may clinically resemble other common lesions, such as basal cell carcinoma.

Histologically, DT is characterized by typical features of trichilemmoma, including basaloid cells, peripheral palisading, and a thickened, eosinophilic basement membrane, in addition to a central desmoplastic stroma, mimicking invasive carcinoma [1,4]. However, the lesion is benign. Notably, atypical basaloid proliferations, including basal cell carcinoma, have been reported in association with DT with an estimated frequency of 9% [3]. We report the case of a patient with a biopsy suggestive of DT; however, upon complete excision of the lesion with Mohs micrographic surgery, superficial basal cell carcinoma was identified. The role of complete surgical excision for DT is discussed, and the utility of Mohs micrographic surgery is highlighted. This article was previously presented as a poster at the Spring 2024 Texas Dermatological Society Conference on April 19, 2024.

## Case presentation

**Figure 1:**
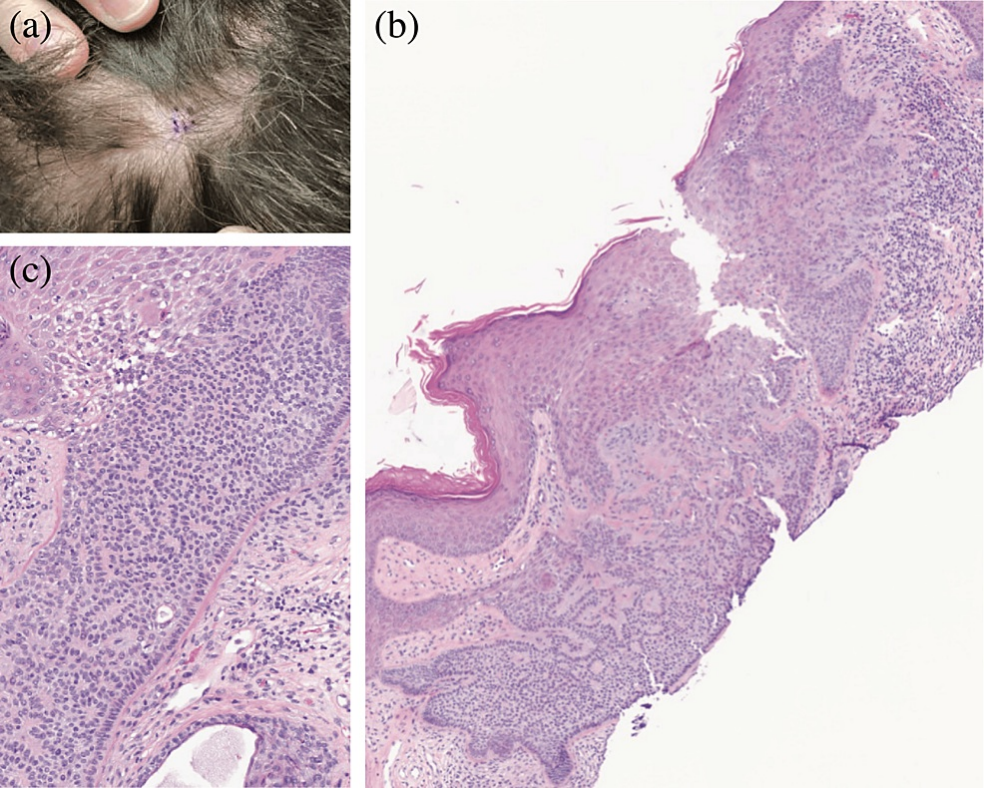
(a) Clinical image of the patient’s lesion, demonstrating a pink, pearly papule on the vertex scalp. (b) Shave biopsy of the patient’s lesion at low power. Note the basaloid appearance, peripheral palisading, and prominent basement membrane. A central desmoplastic stroma is also seen (hematoxylin and eosin (H&E), original magnification x4). (c) Shave biopsy of the lesion at higher power, further demonstrating the peripheral palisading and prominent basement membrane, typical features of trichilemmoma (H&E, magnification x20).

**Figure 2:**
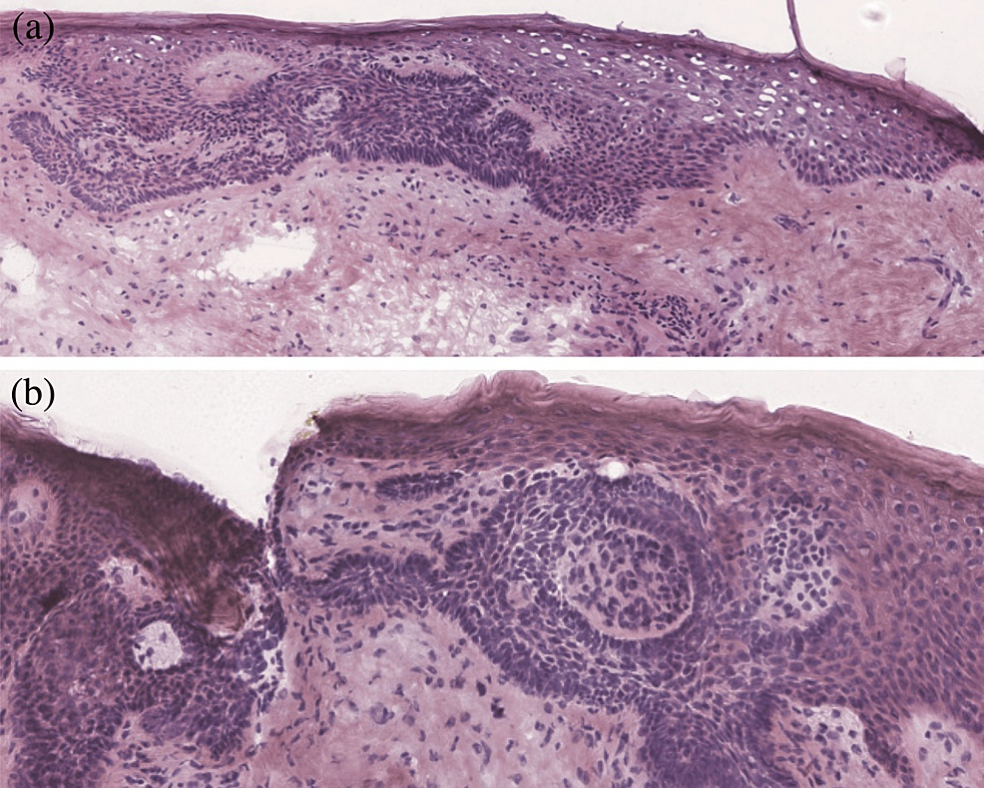
(a) Findings from Stage I of Mohs micrographic surgery (hematoxylin and eosin (H&E), original magnification x10). (b) Note the atypical basaloid cells, hyperchromatic nuclei, scant cytoplasm, and peripherally palisading nuclei in aggregates emanating from the undersurface of the epidermis, consistent with a diagnosis of superficial basal cell carcinoma (H&E, original magnification x10).

**Figure 3:**
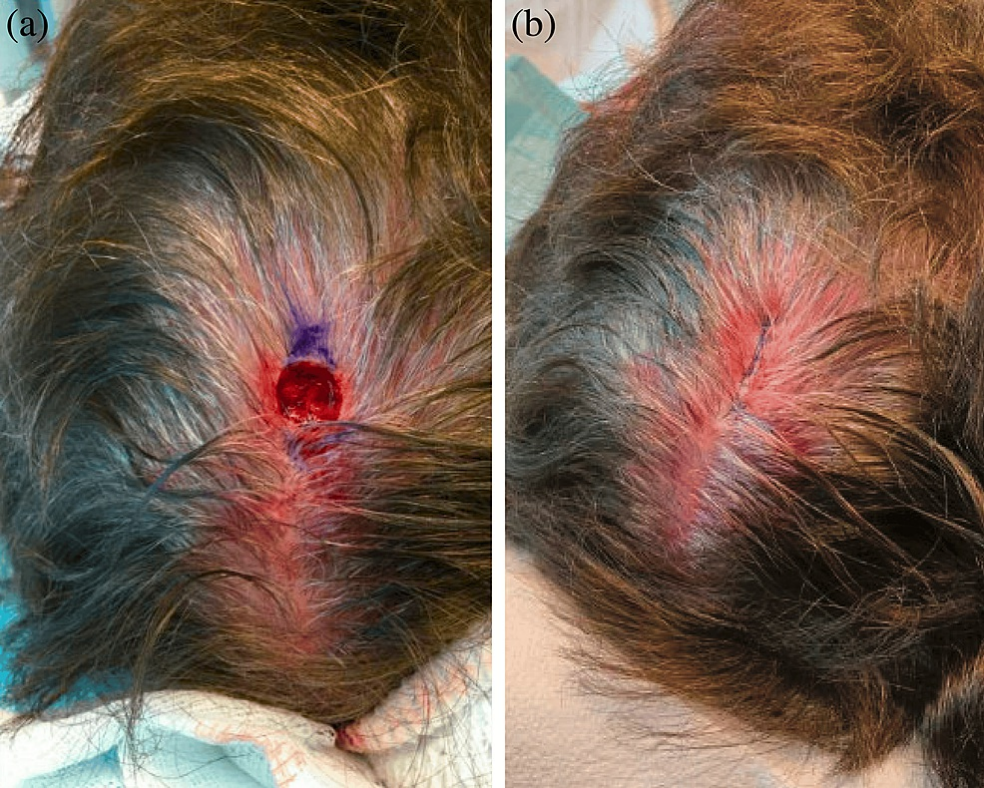
(a) Final defect measuring 1.3 x 1.2 cm after Mohs micrographic surgery. Negative margins were achieved after two stages. (b) Intermediate linear closure of the defect with a final length measuring 3.1 cm.

A healthy 67-year-old female presented to our clinic for a skin check. On physical examination, a 0.5 x 0.4 cm pearly, pink papule was noted on the vertex scalp (Figure 1a). The patient was unsure how long the lesion had been present, so a shave biopsy was obtained to rule out malignancy, which was the primary differential diagnosis. Histopathologic examination of the lesion revealed a basaloid epithelial neoplasm in continuity with the epidermis, a central desmoplastic appearance, and a prominent basement membrane (Figures 1b, 1c). DT was favored based on these typical features; however, the biopsy was incomplete. Given the location of the lesion and the known association of DT with atypical basaloid proliferations, Mohs micrographic surgery was chosen for definitive treatment. Subsequently, upon examination of the Stage I Mohs slides, focal superficial basal cell carcinoma was identified within the lesion (Figures 2a, 2b). Negative margins were achieved after two stages, and the resulting defect was successfully repaired with an intermediate linear closure (Figure 3).

## Discussion

Herein, we reported the case of a patient with an initial shave biopsy consistent with DT; however, superficial basal cell carcinoma was identified upon complete excision of the lesion with Mohs micrographic surgery. Ultimately, the association of DT with basal cell carcinoma is rare, but not negligible, and this case serves to further strengthen this known association. Furthermore, the presence of basal cell carcinoma may not be observed on the initial biopsy of these lesions, as punch and shave biopsy techniques inherently only provide a partial sample of a given lesion.

For these reasons, complete surgical excision for the treatment of DT may prevent a missed diagnosis of basal cell carcinoma or other atypical basaloid proliferations in incompletely sampled lesions. However, close clinical follow-up may also be reasonable if surgical intervention is not feasible, such as in the case of patient preference, for example. Mohs micrographic surgery is an especially useful technique for the surgical removal of DT, as opposed to wide local excision, given its ability for complete margin control with maximal preservation of the surrounding tissue [5].

## Conclusions

We reported a case of DT successfully treated with Mohs micrographic surgery, and in our case, the presence of superficial basal cell carcinoma and positive margins after Stage I further underscores its necessity. Taken together, this case demonstrates the role of complete surgical excision for DT, particularly in incompletely sampled lesions given the potential association with basal cell carcinoma, and highlights the utility of Mohs micrographic surgery for its removal.

## References

[1] McCalmont TH (2012). Adnexal neoplasms. Dermatology.

[2] Crowson AN, Magro CM (1996). Basal cell carcinoma arising in association with desmoplastic trichilemmoma. Am J Dermatopathol.

[3] Afshar M, Lee RA, Jiang SI (2012). Desmoplastic trichilemmoma--a report of successful treatment with Mohs micrographic surgery and a review and update of the literature. Dermatol Surg.

[4] Tellechea O, Reis JP, Baptista AP (1992). Desmoplastic trichilemmoma. Am J Dermatopathol.

[5] Schweiger E, Spann CT, Weinberg JM, Ross B (2004). A case of desmoplastic trichilemmoma of the lip treated with Mohs surgery. Dermatol Surg.

